# Supplementary material for: Learning curve and surgical outcome of robotic assisted colorectal surgery with ERAS program
Source: Sci Rep. 2022 Nov 29;12:20566. doi: 10.1038/s41598-022-24665-w (PMC9709162; doi:10.1038/s41598-022-24665-w)
Supplement: Supplementary file 1 — Supplementary Information 1. [file 41598_2022_24665_MOESM1_ESM.zip › LSC Raw data-Ñ[▒K/060/OP N_ñw╝╨░O▒Kñσ.pdf]

W52 / W52

臺中榮民總醫院
手術室護理記錄

[redacted]

基本資料

- 室別： 開刀房-A5室，手術科別： CRS，手術類別： 常規，日期： 2020/01/09
- 進入時間： 1340 ； 麻醉開始時間： 1345 ； Time Out時間： 1437 ； 劃刀時間： 1439 ； 手術結束時間： 1740 送出時間： 1800 ；
- 麻醉方式： GE
  診斷： Malignant tumor of hepatic flexure
  手術名稱： Laparoscopic right colectomy and anastomosis
  手術局部用藥： 無
  術前預防性抗生素：
  - 抗生素名稱一： Veterin 1GM IVD由麻醉護理師石芳茹給予
  - 時間： 1430
  - 上次簽章: 張巧築

  ，追加預防性抗生素：
- 手術醫師： 蔣鋒帆，林昌霖，楊涵中，陳威遠
- 麻醉醫師： VS 王賜洲
-
  - 刷手護士A ： 陳有容，時間： 1340，接替刷手一： 陳祈安，時間： 1500
  - 流動護士A ： 張巧築，時間： 1340，接替流動一： 陳有容，時間： 1500，接替流動二： 陳家瑋，時間： 1730

皮膚狀況

術前： 完整
記錄者： 上次簽章: 張巧築
術後： 完整
記錄者： 陳家瑋

手術準備

手術臥式 單一手術臥式

-
  - 手術臥式： 仰臥
  - 使用附件： 溫毯 38 ℃，頭圈，其它： 頭架、矽膠墊
  - 皮膚準備： 10%Aq Betadine，Hibiscrub，其它： 2% Chlorhexidine
  - 電燒： 使用電燒: 電極片位置： 右小腿，記錄者： 上次簽章: 張巧築

止血帶T： 無

植入物

無

標本送檢

- 細菌培養 無
- 細胞學 無
- 冰凍切片 無
- 病理標本
  組織名稱一： Right side colon
- 截肢 無

標本交付 標本： 1 袋，標本： 0 盒，標本： 0 瓶，細胞學： 0 管，Culture Tube： 0 管，被交付醫師： R 楊涵中，

- 記錄者： 陳家瑋
- 時間： 1740

傷口術後敷料

傷口敷料：　　　　　　　　　　　　傷口敷料： 無

- 請於圖上標示部位：

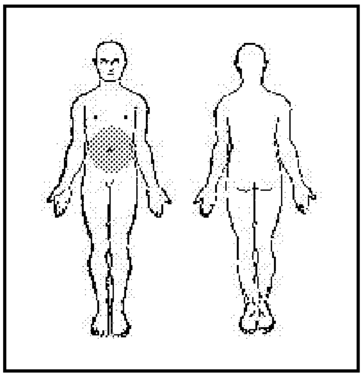

- 傷口敷料： 軟性敷料。

導管

導管	天數	導管資訊	插入日期	插入時間	更換日期	拔除日期	拔除時間	拔除原因	預計拔除/更換	預計拔除/更換日期
Penrose Drain(1)	1	號碼:12 mm.; 部位:肝下;	2020/01/09	1700						
A-line (1)	1	號碼:20 G; 部位:右手;	2020/01/09	1400						
Foley(1)	1	號碼:16 Fr.; 部位:膀胱; 註記:Balloon 10cc;	2020/01/09	1420						
Sump NG Tube(1)	1	號碼:16 Fr.; 深度:55cm; 部位:胃;	2020/01/09	1440						

用物計數

- 手術中出血量： MINIMAL c.c.
- 手術中尿量： NIL c.c.
-
    - 其它： 無

用物計數： 多切口同時縫合

- 1.器械： 正確，2.縫針： 正確，3.刀片： 正確，4.紗布： 正確，5.腹部墊： 未使用，6.紗球： 未使用，7.PEANUTS ： 未使用，8.紗條： 未使用，9.絲帶： 未使用，10.針頭： 未使用，11.Vessel loop： 未使用，12.Boots： 未使用，13.Jelco： 未使用，14.棉條： 未使用，
    - 第一次：
        - 刷手護士 ： 陳祈安
        - 流動護士： 上次簽章: 陳有容
        - 時間： 1700

  ,
    - 第二次：
        - 刷手護士 ： 陳祈安
        - 流動護士： 上次簽章: 陳有容
        - 時間： 1710

  ,
    - 第三次：
        - 刷手護士 ： 陳祈安
        - 流動護士： 上次簽章: 陳有容
        - 時間： 1715

備註

備註： 腹腔鏡2號

- 記錄者： 上次簽章: 張巧築
- 時間： 1455

填寫護理人員 陳家瑋

正式摘要

W52 / W52

doctorRank:B
